# Acoustic speech features in social comparison: how stress impacts the way you sound

**DOI:** 10.1038/s41598-022-26375-9

**Published:** 2022-12-20

**Authors:** Mitchel Kappen, Jonas van der Donckt, Gert Vanhollebeke, Jens Allaert, Vic Degraeve, Nilesh Madhu, Sofie Van Hoecke, Marie-Anne Vanderhasselt

**Affiliations:** 1 Department of Head and Skin, Department of Psychiatry and Medical Psychology, Ghent University, University Hospital Ghent (UZ Ghent), Corneel Heymanslaan 10-13K12, 9000 Ghent, Belgium; 2 Ghent Experimental Psychiatry (GHEP) Lab, Ghent University, Ghent, Belgium; 3 Department of Experimental Clinical and Health Psychology, Ghent University, Ghent, Belgium; 4 IDLab, Ghent University-Imec, Ghent, Belgium; 5 Department of Electronics and Information Systems, Ghent University, Ghent, Belgium

**Keywords:** Psychology, Biomarkers

## Abstract

The use of speech as a digital biomarker to detect stress levels is increasingly gaining attention. Yet, heterogeneous effects of stress on specific acoustic speech features have been observed, possibly due to previous studies’ use of different stress labels/categories and the lack of solid stress induction paradigms or validation of experienced stress. Here, we deployed a controlled, within-subject psychosocial stress induction experiment in which participants received both neutral (control condition) and negative (negative condition) comparative feedback after solving a challenging cognitive task. This study is the first to use a (non-actor) within-participant design that verifies a successful stress induction using both self-report (i.e., decreased reported valence) and physiological measures (i.e., increased heart rate acceleration using event-related cardiac responses during feedback exposure). Analyses of acoustic speech features showed a significant increase in Fundamental Frequency (F0) and Harmonics-to-Noise Ratio (HNR), and a significant decrease in shimmer during the negative feedback condition. Our results using read-out-loud speech comply with earlier research, yet we are the first to validate these results in a well-controlled but ecologically-valid setting to guarantee the generalization of our findings to real-life settings. Further research should aim to replicate these results in a free speech setting to test the robustness of our findings for real-world settings and should include semantics to also take into account what you say and not only how you say it.

## Introduction

Stress is omnipresent in modern society. Whereas low levels of stress can increase one’s performance, the chronic experience of stress is a common risk factor for a variety of different mental and physical health problems^1,2^, making it a critical factor in determining human health^3^. Therefore, frequent, accurate, and affordable stress measurement tools would be of great contribution to society as regular observation of increased acute stress levels would be indicative of a chronically stressed state.

Acute stress, in scientific studies, is often measured by using either self-report assessments or monitoring physiological signals such as electrocardiography or electrodermal activity^4^. However, the use of speech as a novel biomarker for (acute) stress has rapidly gained attention due to it being non-intrusive (no physical connection necessary to the body), affordable to acquire, and ubiquitous, considering the increasing presence of high-quality microphones in everyday objects^5,6^. As stress influences important factors in speech production such as breathing, cardiac activity, or general pose, it is hypothesized that experienced stress could be detected from acoustic features of one’s voice (for an extensive explanation of each step of speech production with regards to these features, see: Van Puyvelde et al.^7^). Moreover, speech has increasingly shown to be a potential sensitive marker for depression, schizophrenia, and autism^8–10^. Since stress is considered a key underlying working mechanism of negative mood and a risk factor for the development of mood disorders and the expression of a wide range of psychological diseases, its effects on speech could further progress the (early) detection of numerous psychological diseases. In addition, stress could affect what words you utter, their complexity, and other prosodic features due to changes in cognitive load^11,12^. However, to isolate and evaluate the effects of stress on acoustic speech features, it is necessary to exclude both interindividual differences in linguistic capabilities and acoustic effects induced by variations in words and sentences by using read-out-loud speech fragments before moving on to include linguistic features such as syntax and semantics.

As the field of measuring stress in speech is evolving quickly, it is proposed that the vocal response to stress may be as individual and unique as the voice itself, and thus more isolated studies that control for interindividual differences are required^5,7^. Whereas many recent studies use large samples of audio fragments and extract a wide scale of features using easily accessible toolboxes (e.g. PRAAT, OpenSMILE^13,14^), some limitations can be noted. It is argued that these studies (1) are often between-subject, therefore unable to contain interindividual differences in the stress response, (2) lack a valid verification of the subject’s emotional state, or (3) include a wide range of (acoustic) features that lack scientific basis, which increases the risk of overfitted models that would not generalize well to everyday life situations^5,7,15^.

Psychosocial stressors are one of the most potent and ecologically-valid stressors and are induced in situations of social evaluation or exclusion^15–17^. Our former study used a similar paradigm, which confirmed the stress induction was successful, but the study was limited to pre-, and post-stressor measurements, thus lacking a control/neutral condition^15^. In our current study, a successful stress induction will be determined based on both self-reports (valence, arousal) throughout the paradigm and physiological activity (cardiac acceleration and deceleration) during the negative versus the control feedback exposure. Therefore, we believe we contribute to the existing literature by (1) a matter of developing a new psychophysiological methodology for stress measurement, and (2) do so by designing a solid experimental paradigm that allows us to induce and validate experienced stress on an individual (within-participant) basis.

Despite mixed results in acoustic changes due to acute stress, some acoustic features get described more often than others in literature. As such, we will focus on these key acoustic speech features from literature in the current study. The most homogeneous results are found for the Fundamental Frequency (F0) of the voice, which refers to the frequency at which the vocal cords vibrate (i.e. pitch), seeing it generally increases with increased stress^5,7^. We expect to find decreases for jitter (vocal frequency variation) and shimmer (vocal intensity variation) as that is the direction of observed effects, however, results are heterogeneous^5,7^. Harmonics-to-noise ratio (HNR; added noise in the voice) has been shown to decrease in the context of a physical stressor (i.e., workout), and mixed results are observed in the context of psychological stress^5,18–20^. We included HNR as it is frequently described in the literature and changes are observed, however, we have no expected direction for this effect. Lastly, we expect participants’ speech rates to increase. This feature is not always included in analyses but has shown robust results in free speech settings^5,21,22^.

In summary, in the presented study in this paper, we analyze high-quality read-out-loud speech fragments collected from a large (non-actor) sample in a within-subject stress paradigm, containing both a control and a negative feedback condition. In doing so, we can verify the experienced stress by the subjects (based on both self-reports and objective physiological measures). We present trustworthy and ecologically valid information on the distilled effects of (psychological) stress on key acoustic speech features, such as F0 (Fundamental Frequency; pitch), jitter (vocal frequency variation), shimmer (vocal intensity variation), harmonics-to-noise ratio (HNR; added noise in the voice), and speech rate^5,7,15^. These results will be the basis for further deterministic modeling, digital biomarker design, and/or analysis of speech in the context of stress detection and emotion recognition. Moreover, these results will contribute to the field of vocal markers of neuropsychiatric conditions, considering stress is suggested to be a core psychological mechanism associated with a range of mood disorders e.g. see^8–10^.

## Results

### Manipulation check

A manipulation check was conducted to verify whether participants experienced increased stress during the negative feedback condition compared to the control feedback condition by setting side-by-side self-reports and physiological activity during both feedback conditions.

#### Self-reports

**Figure 1:**
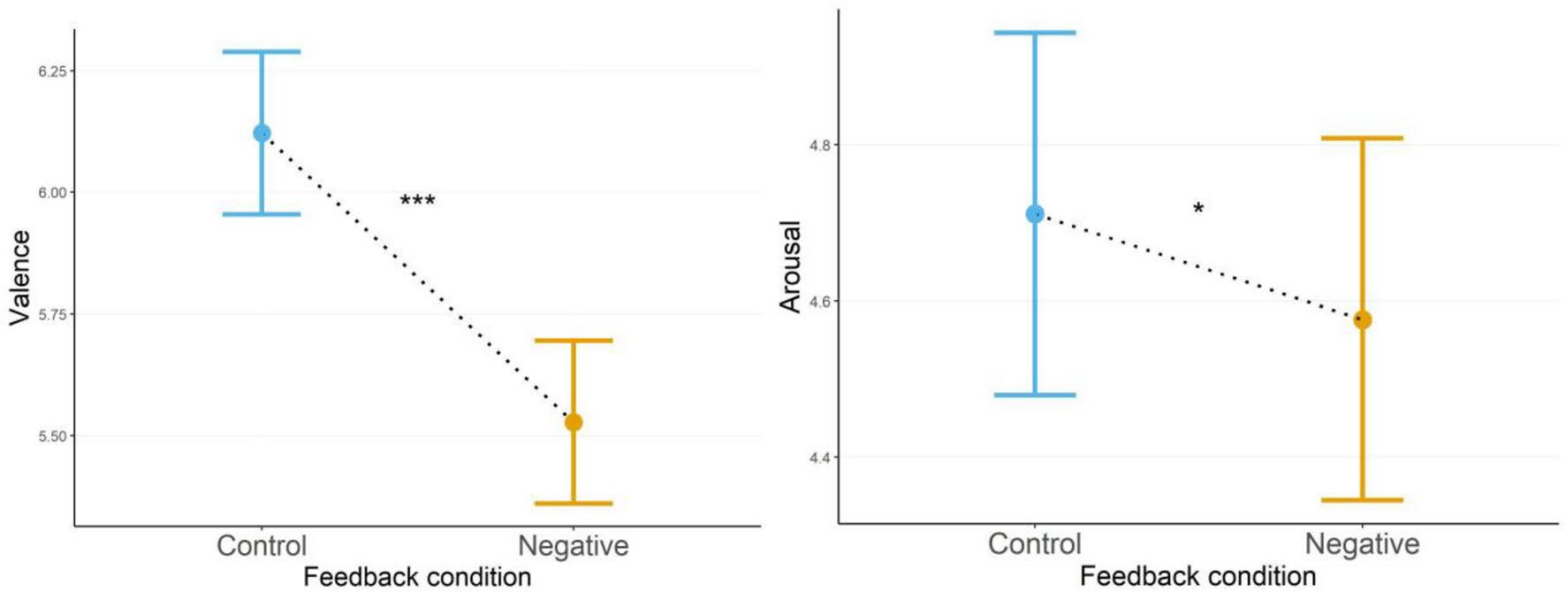
(**a**) Valence between feedback conditions. (**b**) Arousal between feedback conditions. Estimated marginal means (EMMs) of self-reported valence (**a**) and arousal (**b**) during control-, and negative feedback condition after controlling for sex. Error bars depict standard error of the means (SEMs), asterisks indicate significance levels. *p < 0.05. ***p < 0.001.

At three different set moments during each condition, participants answered how they were feeling with regards to *valence* and *arousal* using SAMs (Self-Assessment Manikins). Valence, with the formula *Valence* ~ Condition + *(1|ID)*, was best described by an LMM (linear mixed model) with AIC equal to 1055 (Akaike Information Criterion). The LMM showed a significant decrease of valence in the negative feedback condition (Fig. 1a) *χ*^2^(1) = 83.01, *b* = 0.594, *SE* = 0.0652, *t* = 9.111, *d* = 0.91, *p* < 0.001. Arousal, with the formula *Arousal* ~ *Condition* + *(1|ID)*, was best described by a GLMM (generalized linear mixed model) with Gamma distribution and identity link, AIC = 1069. The GLMM showed a significant decrease in arousal during the negative feedback condition (Fig. 1b) *χ*^2^(1) = 4.47, *b* = 0.135, *SE* = 0.0639, *z* = 2.116, *d* = 0.50, *p* = 0.034.

#### Physiological activity

**Figure 2:**
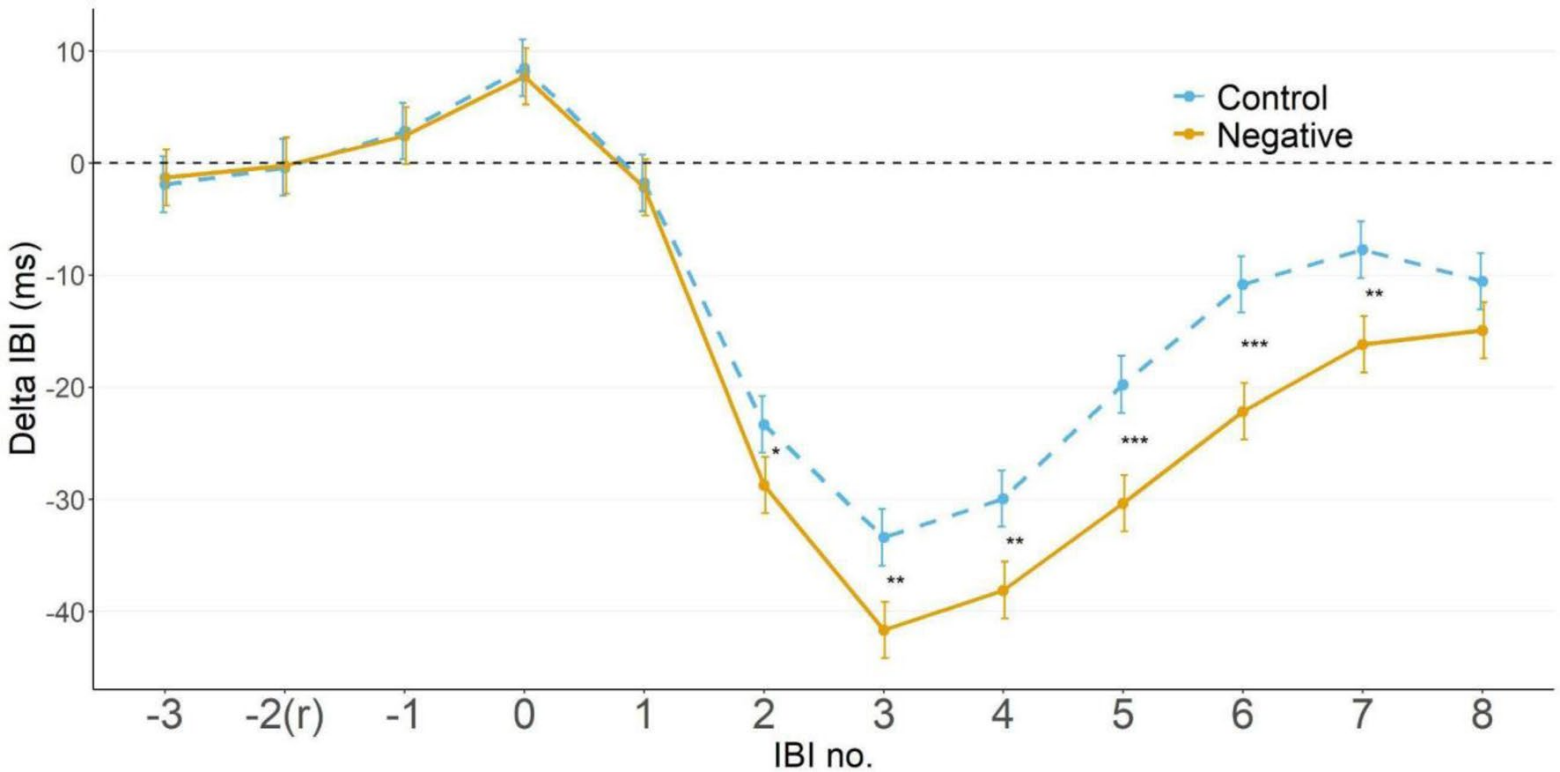
Delta Interbeat intervals in response to feedback exposure between feedback conditions. Estimated Marginal Means (EMMs) for delta IBI’s (in ms, referenced to IBI_-2_) of control-, and negative-feedback trials, with IBI_0_ being IBI closest to feedback exposure onset. Error bars depict the standard error of the means (SEMs), asterisks indicate significance levels.*p < 0.05. **p < 0.01. ***p < 0.001.

Delta IBI’s (cardiac interbeat interval) were calculated during the feedback period (6 s) after a trial was completed. Considering the presence of non-positive values (cardiac acceleration: negative delta’s), an LMM with the formula *DeltaIBI* ~ *Condition × IBI*_*no*_ + *(1|ID)* was fit to the data. The LMM showed a Condition x IBI_no_ interaction effect *χ*^2^(11, *N* = 73) = 33.49, p < 0.001, showing more acceleration in heart rate during observation of the negative feedback than the control feedback. However, as our main focus is on the IBIs following feedback exposure rather than on all IBIs; follow-up pairwise comparisons were executed between the two conditions at every individual IBI, on which we applied FDR (False Discovery Rate) correction^23^. We observe significant effects for IBI_2_ to IBI_7_ (Fig. 2, Table 1), showing that heart rate acceleration is larger during exposure to negative feedback as compared to control feedback from IBI_2_ to IBI_7_ (see Table 1).

**Table 1:** Individual contrasts at different IBIs between control-, and negative-feedback condition trials.

	*b*	*SE*	*z*	*p*
IBI_0_	0.760	2.58	0.295	0.770
IBI_1_	0.393	2.58	0.153	0.879
IBI_2_	5.381	2.58	2.089	0.037
IBI_3_	8.282	2.58	3.215	0.001
IBI_4_	8.181	2.58	3.176	0.002
IBI_5_	10.574	2.58	4.105	< 0.001
IBI_6_	11.301	2.58	4.388	< 0.001
IBI_7_	8.437	2.58	3.276	0.001
IBI_8_	4.356	2.58	1.691	0.091

*b* is the beta coefficient, SE is the standard error of the difference, *z* is the z-ratio, *p* is the p value. p values are FDR corrected.

### Speech feature analysis

For each of the speech features, a series of (G)LMM (generalized linear mixed models) were fitted to increase the likelihood of using a statistical model that best fits the underlying distribution. Model selection was performed using the AIC. To minimize the likelihood of Type 1 errors, FDR correction was applied over all p values for the different speech features.

#### Harmonics-to-noise ratio (HNR)

**Figure 3:**
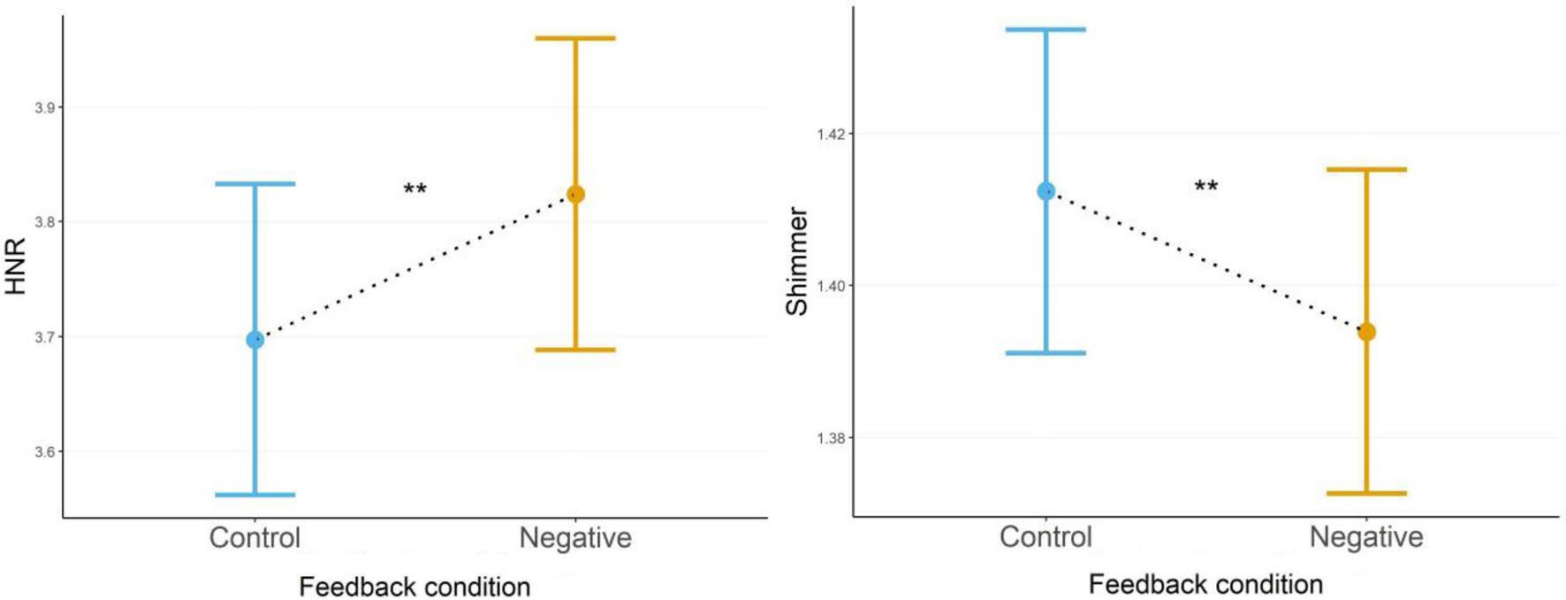
(**a**) HNR between feedback conditions. (**b**) Shimmer between feedback conditions. Estimated marginal means (EMMs) of HNR (**a**) and Shimmer (**b**) during control-, and negative feedback conditions after controlling for sex. Error bars depict standard error of the means (SEMs), asterisks indicate significance levels. **p < 0.01.

The distribution for HNR was best represented by an LMM (AIC = 765) and showed a significant main effect for the feedback condition after controlling for sex, with HNR being significantly higher during the negative-, versus the control-feedback condition, *χ*^2^(1) = 8.17, *b* = 0.127, *SE* = 0.0444, *z* = 2.858, *d* = 0.68, *p* = 0.005 (Fig. 3a).

#### Shimmer

Shimmer was best represented by a GLMM with Gamma distribution and identity-link (AIC = − 927) and showed a significant main effect for the feedback condition after controlling for sex, with shimmer being significantly lower during the negative-, versus the control-feedback condition, *χ*^2^(1) = 8.30, *b* = 0.019, *SE* = 0.006, *z* = 2.881, *d* = 0.68, *p* = 0.004 (Fig. 3b).

#### Fundamental frequency (F0)

**Figure 4:**
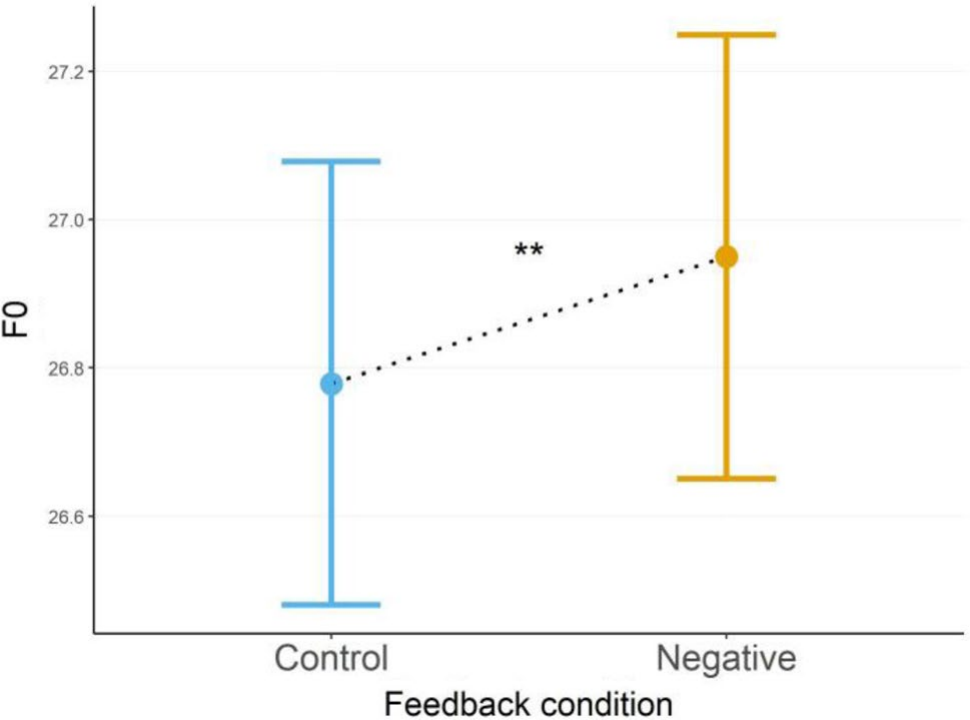
Fundamental Frequency (F0) between feedback conditions. Estimated marginal means (EMMs) of Fundamental Frequency (F0) during control-, and negative feedback conditions after controlling for sex. Error bars depict standard error of the means (SEMs), asterisks indicate significance levels. **p < 0.01.

F0 was best represented by a GLMM with Gamma distribution and identity-link (AIC = 961) and showed a significant main effect for the feedback condition after controlling for sex, with F0 being significantly higher during the negative-, versus the control-feedback condition, *χ*^2^(1) = 7.60, *b* = 0.171, *SE* = 0.062, *z* = 2.756, *d* = 0.65, *p* 0.006 (Fig. 4).

#### Jitter, voiced segments per second, mean voiced segment length

No significant effects were observed for either jitter, voiced segments per second, or mean voiced length. Jitter was fit with an LMM (AIC = − 2725), *χ*^2^(1) = 0.975, *p* = 0.32. Voiced segments per second were fit with a GLMM with Gamma fit and identity link (AIC = 20), *χ*^2^(1) = 2.41, *p* = 0.12. Mean voiced segment length was fit with a GLMM with Gamma fit and identity link (AIC = − 2002), *χ*^2^(1) = 3.25, *p* = 0.07.

## Discussion

In this study, we aimed to examine the effects of stress, induced by a highly controlled social evaluative threat stressor, on different key speech features (fundamental frequency; F0, harmonics-to-noise ratio; HNR, jitter, shimmer, and speech rate). Participants performed tasks within two conditions, one with control feedback and one with negative comparative feedback, that shared the same overall design. Each feedback condition contained three subblocks of abstract reasoning puzzles (i.e., Raven’s matrices) to be solved under time pressure. In the first condition, participants were told that they were being compared to a group of people who were randomly sampled from the population (average individuals) and received feedback after each trial that indicated that they were performing on par with the reference group (control feedback condition). In the second condition, participants were told that they were being compared to individuals that achieved significant academic or professional success (to increase the credibility of a sudden drop in relative performance), and received feedback after each trial, indicating that they were performing increasingly worse compared to the comparison group throughout each subblock (negative feedback condition). During each condition, participants were asked at three moments to read a standardized text out loud. We extracted several key acoustic features from these fragments to gain insight into the effects of acute (psychosocial) stress on speech. These features were selected based on previous research in which they were deemed important in the context of stress^5,7,15^. However, this study is the first to use a within-participant design that verifies a successful stress induction using both self-report and physiological measures in a (non-actor) sample. We verified a successful stress induction based on decreased self-reported valence scores and increased heart rate acceleration during the negative feedback condition. Increased heart rate acceleration during negative feedback is consistent with the notion of increased sympathetic reactivity due to stress exposure^24,25^. However, we did encounter a decrease in self-reported arousal during the negative feedback condition, which is the opposite of what we expected. This result could potentially be an order effect, such as tiredness, due to the negative feedback condition always being subsequent to the control condition. Nonetheless, we conclude a successful stress induction due to the increased heart rate acceleration and decrease self-reported valence during the negative feedback condition.

We observed an increase of F0 (fundamental frequency; pitch) during the negative feedback condition as compared to the control feedback condition. This was expected since increases in F0 in response to an acute (psychosocial) stressor are commonly reported in the literature^5,7^. Furthermore, we observed a significant increase in HNR (harmonics-to-noise ratio; added noise in the voice) in the negative feedback condition as compared to the control feedback condition. In the past, no clear results have been found with regard to this parameter, as it has shown to decrease in the context of physical stress tasks (e.g., workout) and has shown mixed results in the context of cognitive load/psychological stress^5,17–19^. We also found a decrease in shimmer (vocal intensity variation) during the negative as compared to control feedback. The effects of stress on shimmer are less pronounced, where some studies indicate no changes and others a decrease in shimmer after different stress induction procedures^5,20^. Nonetheless, we found a clear decrease in shimmer during stress, which could make sense due to its vowel-level relationship with heart rate, a central component in stress reactivity^5,26^. Yet, future research should revisit the direct, trial-based relationship between shimmer heart rate.

No effects have been found for jitter (vocal frequency variation). However, as proposed by Van Puyvelde et al.^7^, acoustic speech parameters should not only be considered in their own regard but also as combined patterns of multiple speech parameters that may respond in a simultaneous manner. HNR has been demonstrated to be more sensitive to subtle differences in vocal function than jitter^5,27^ and former network analysis has shown a strong negative relationship between changes in jitter and changes in HNR after psychosocial stress induction^15^. Moreover, jitter is mainly affected due to a lack of control of the vocal fold vibration^28^. The lack of a significant difference could be explained by the nature of the speech fragments that we analyzed. It could be argued that when people read a text out loud, as opposed to speaking freely, they could be using a ‘reading voice’ that minimizes these types of effects due to read speech being significantly different from spontaneous speech, both acoustically and linguistically^29^. A similar argument could be made to explain a lack of effect found for the speech rate, as when someone reads out loud, one of their focuses is understandability for potential listeners^29^. In addition, since the text and one’s ability to process this both influence a minimal and maximum speech rate, it can be expected that this measure is limited by the speech recording paradigm.

The current results are generated in a well-controlled experimental setting, using a stressor with a control condition that also contains time pressure. Therefore, the presented results are indicative of how speech as a biomarker reacts to actual stress as induced by negative evaluation, rather than cognitive load or time pressure. However, it should be noted that the current study was limited in its design as the stress condition was always preceded by the control condition rather than being randomized. We chose to use this design because it would enable us to integrate an active control condition which also contained cognitive load and time pressure just as the stress condition, isolating the results to just the experience of negative evaluation. Counterbalancing the order of the control and stress condition would have only been possible by either severely lengthening the design or by testing on multiple days, due to the duration of the recovery phase after a stressor. This limitation could introduce order effects, such as a fatigue or repetition effect, that confound our presented results. Nevertheless, considering our results are in line with previous research, we believe that if this effect influenced our results, they potentially reduced the observed effect sizes. Supplemental analyses indeed show no effect of repetition on the speech features (https://osf.io/gq7aw).

The current study evaluated several key acoustic speech features in an isolated situation; by using read-out-loud speech, potential interference from specific word choices was eliminated. However, to work towards a real-world application for speech as a biomarker for stress, features in spontaneous speech fragments should also be tested. Future studies should therefore move towards a speech collection paradigm that enables participants to speak freely. However, certain considerations should be made here, since completely free speech would introduce a number of noise factors that could make the comparison of certain acoustic features between different conditions close to impossible. In order to more closely simulate free speech in a controlled setting, future research should focus on using a speech collection paradigm in which participants are semi-spontaneous in their speech by, for example, controlling the topics they can talk about, whilst limiting any extra cognitive load of active recall. Shifting towards free speech should give us more insight into the robustness of these acoustic speech features in more spontaneous settings, and will additionally enable us to investigate other linguistic features of speech in the context of stress, such as syntax, prosody, and semantics. Combining semantics with syntax and acoustic features will indicate the potential of real-life applications for stress monitoring using speech signals. Moreover, we invite others to use our data and test other features that they deem promising (https://osf.io/78g9s/).

To conclude, we collected repeated read-out-loud speech fragments of participants in a social evaluative threat stress induction paradigm which we validated through self-reports and psychophysiological responses. We were able to give valid and reliable results for the effects of psychosocial stress on F0, HNR, and shimmer, and were not able to find effects on jitter and speech rate. Therefore, we conclude that changes in F0, HNR, and shimmer are shown to be present in speech after stress irrespective of a person’s language construction capability. As such, this study shows that speech is a promising biomarker for stress, on top of it being affordable, non-intrusive, and easy to collect and therefore easy to implement in everyday settings. Future studies should focus on replicating our findings to test the robustness of the effect of stress on these acoustic speech features. In addition, different speech production paradigms should be developed and tested in order to move towards more spontaneous speech and test the external validity in more naturalistic settings. Lastly, this would also enable us to increase the range of speech features that can be informative in the context of stress, such as semantics and syntax.

## Methods

This study was part of a larger project that investigates the effects of a (psychosocial) stressor on neural correlates. Results of electrophysiological correlates will be published elsewhere. Moreover, collected data that was not part of the current paper’s research objectives will only be described in the Supplemental Materials.

### Participants

A convenience sample of 77 subjects (50 female, 27 male, age *M* = 23.13, *SD* = 6.19) was recruited through social media with a post containing the cover story that this study gauges future (academic) success. Upon registration, participants were checked for exclusion criteria (see Supplemental Material). The study was conducted in accordance with the declaration of Helsinki and received ethical approval from the Ghent University hospital ethical committee (registration number: B670201940636). All participants gave written informed consent before participating and were debriefed afterward on the true purpose of the study. A 30 Euro compensation fee was awarded upon completion through bank transfer.

### Apparatus and procedure

#### Read-out-loud text “Marloes”

TO collect speech fragments, participants were instructed throughout the experiment to read a standardized text of five sentences out loud. This “Marloes” text is often used in Dutch speech therapy due to the text containing a similar frequency distribution as occurs in the Dutch language (Van de Weijer & Slis, 1991; See Supplemental Material for full text). Participants were instructed to read the text out loud five times at home prior to the experiment to familiarize themselves with it and to exclude novelty effects^15^.

#### On-site experimental session

The experiment was conducted in a dedicated room in the Department of Neurology at the Ghent University Hospital. The ECG (ElectroCardioGram) electrodes were applied (1 electrode just below the left collarbone and 1 electrode on the left lower rib), after which the experimental phase commenced. Participants were seated in an upright position in front of a computer screen (Dell E2216H).

**Figure 5:**
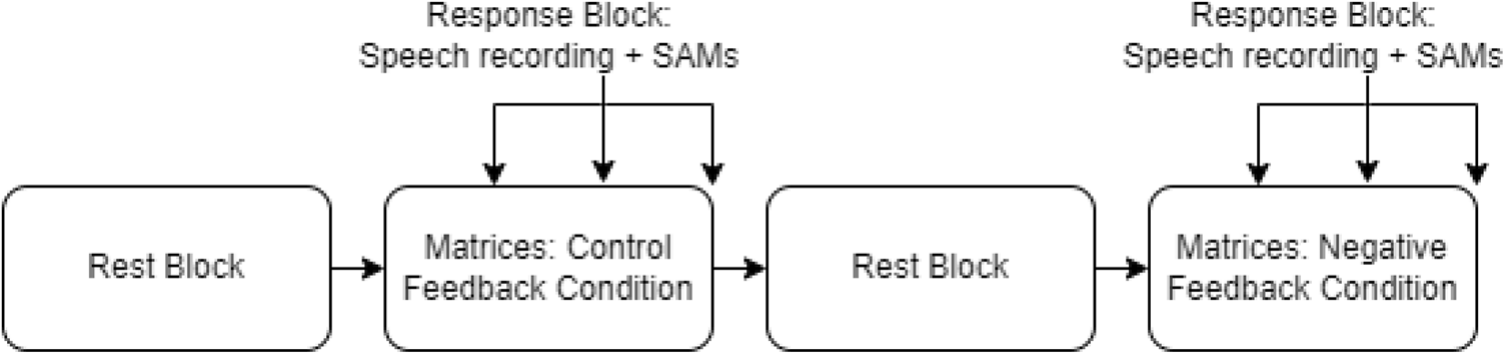
Flowchart of experimental design. Two feedback conditions (control/negative), both preceded by a 10-min resting period. Speech and self-reports (SAMs; Self-Assessment Manikins) were recorded at three points in each condition; yielding 3 data points per participant per condition.

The experiment was carried out on a computer (Dell, Windows 10, experiment designed in E-Prime 2.0^30^ and a tablet (Huawei MediaPad M5, custom-designed Android app; see https://osf.io/78g9s/). The experimental task was completed on the computer, while self-reports and speech collection were done on the tablet to circumvent any built-in preprocessing of the audio signals in E-Prime 2.0. The experiment started with a 10-min resting block (to achieve habituation) in which participants closed their eyes to ensure a relaxed state. After this, the *Control* feedback condition commenced. After this condition, there was another 10-min resting block, followed by a *Negative* feedback condition (see Fig. 5). At three fixed points throughout the two feedback conditions, i.e. at one-third, two-thirds of the way, and at the end of the condition, participants were offered a *Response Block*. The *Response Block* was executed on the tablet and starts with the out-loud reading of the “Marloes” text. After this, participants answered Self-Assessment Manikin scales (SAMs; Valence, Arousal; see Supplemental Material)^31^ by responding which manikin best represented their feelings. Valence was described as how negative/positive they felt at that instance, whereas arousal was described as how calm/restless they felt^32^.

### Trial

**Figure 6:**
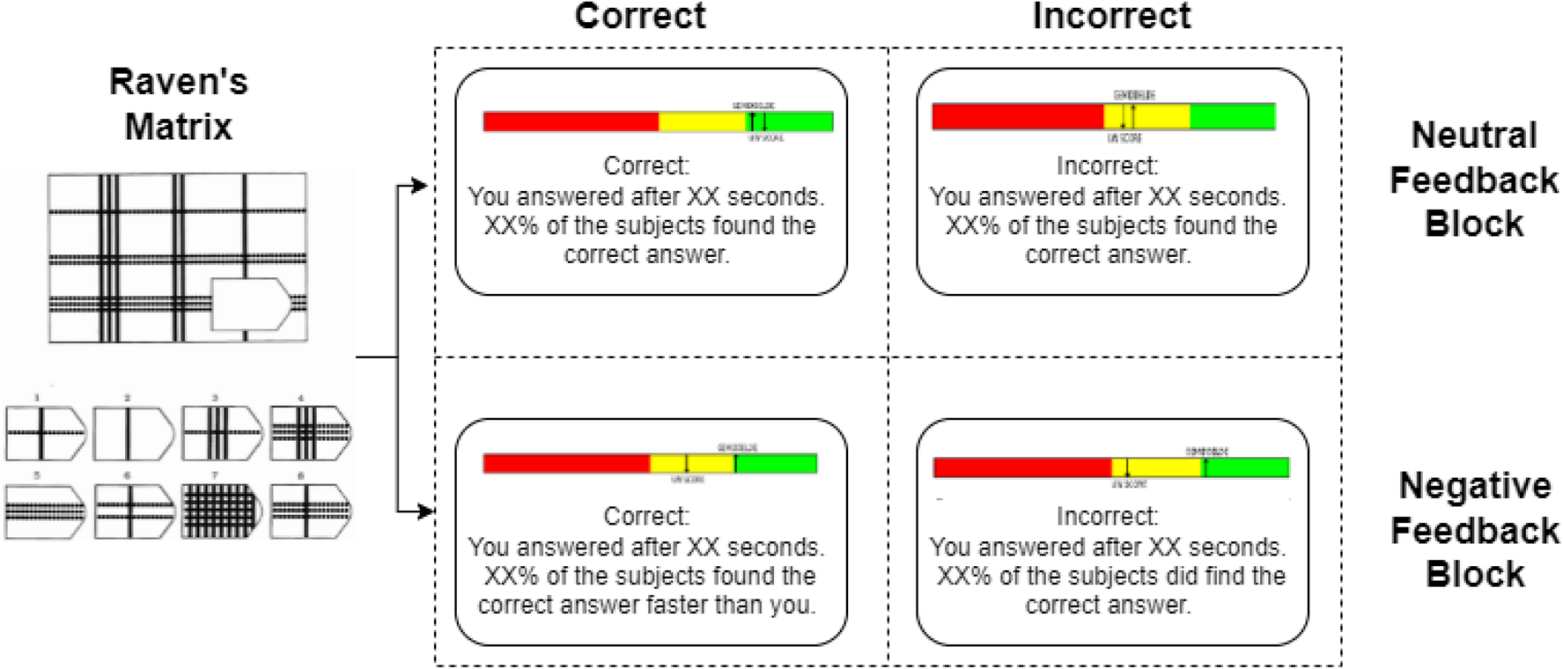
A flow diagram of the trial sequence. Flow diagram of a trial sequence including both options (correct/incorrect) for both conditions (control/negative feedback) after a response to a Raven’s Matrix. On the colored bar, two arrows indicated their own performance and the comparison group performance (accompanied by text) which shifted after every trial. The displayed colors were indicators of overall performance. Matrices varied per condition but were balanced in difficulty over feedback conditions and subblocks.

During each (experimental) feedback condition (control and negative), participants were offered three subblocks. Each subblock ended when participants either completed 11 trials or 6 min had passed since the start of that subblock. During each trial, participants were offered a Raven’s Matrix^33^: a 3 × 3 raster of illustrations that follows a certain pattern/logic with one spot open which they had to fill in (see Fig. 6 for a visual representation of the trial-feedback sequence). Participants could choose from 8 different options to fill in the blank spot and reply with their right hand using the Numpad on a regular US-layout keyboard. Above the raster, a countdown was shown indicating the number of seconds left before the exercise timed out. The allowed time differed per trial and was dependent on the difficulty level; participants had either 20 (for easy levels), 45 (for medium levels), or 100 s (for difficult levels) to respond. The difficulty level was validated in a pilot test to have it balanced over subblock as well as over experimental (feedback) condition and randomized per participant. After either a response or a time-out, a feedback screen was shown. The feedback screen consisted of three elements: (1) at the top, a red, yellow, and green bar was displayed with vertical arrows indicating their performance and the average group performance; (2) it was indicated whether their answer was correct or incorrect (or timed-out), this was always in accordance with their actual answer; and (3) at the bottom of the screen, their response time was indicated as well as a textual comparison to the reference group. This paradigm was inspired by the Montreal Imaging Stress Task (MIST^34^, but we used Raven’s Matrices rather than mathematical puzzles to reduce the stress that is experienced by people who are not good at math, and because something more similar to an IQ test would fit better in the cover story. In addition, the time pressure did not vary between the control and negative feedback conditions in our paradigm, shifting it from a cognitive stressor to a psychosocial stressor.

### Stress induction

Both (experimental) feedback conditions were essentially identical, except for the comparison group and the received feedback. During the control feedback condition, to increase the credibility of the cover story, participants are told that they are being compared to a sample of people who are randomly sampled from the population (average individuals). In order to successfully induce stress in a repeated MIST paradigm, there must be a credible social evaluation. As such, a credible cover story as used here contributes to the ecological validity of the stressor^35^. When seeing the feedback, they are shown to be performing on par with the reference group to ensure control/neutral feedback. During the negative feedback condition, participants are told that they are being compared to a group of highly educated, well-performing individuals. When observing the colored feedback bar, they are shown to be performing increasingly worse over the course of each subblock, irrespective of their actual performance to enable the negative feedback (see Supplemental Material for a visual representation of increasingly negative feedback over subblocks). Moreover, whenever the participant found the correct answer, the feedback would indicate that XX% of the reference group found the correct answer faster than them, still inducing a negative comparison even when they did give a correct answer. Feedback was displayed for 6 s, after which the next trial commenced. See Fig. 6 for a flow matrix of a trial sequence including examples of the different feedback types for both correct and incorrect responses in both the control and negative feedback conditions. Prior to the experimental task, participants are told a cover story that the study’s objective is to predict future success and that this task was commonly used in IQ tests and was valid in this prediction.

### Extraction of speech features

All audio fragments were manually checked for quality, and only full and clear (i.e., no excessive clipping or background noise) recordings were included. 31 control and 34 negative feedback recordings were of insufficient quality and were not used in subsequent analyses, resulting in 209 control feedback recordings of 71 out of 77 participants and 206 negative feedback recordings of 69 out of 77 participants. Features were extracted using OpenSmile 2.3.0^14^ with the GeMAPS configuration^36^, a minimalistic acoustic feature set frequently used in voice research and affective computing. From this feature set, Fundamental frequency (F0), Jitter, Shimmer, Harmonics-to-Noise Ratio (HNR), voiced segment length, and mean voiced segments per second (a proxy for speech speed) were selected. It is important to remark that the features were computed locally via a sliding-window and then mean-aggregated over the whole utterance, thus not displaying high temporal changes. For detailed information regarding feature calculation and extraction procedure, we refer the reader to Eyben et al.^14^ and Section 6.1 of Eyben et al.^36^.

### Data-analysis

All data were preprocessed using Python 3.9.6 and statistical analyses were performed using R4.1.1 (for detailed version information of the software and packages used, see Supplemental Materials). As a part of our manipulation check, we collected ECG data throughout the task and analyzed the event-related cardiac reactivity during feedback exposure (see for a similar approach: Gunther Moor et al. 2010; van der Veen et al. 2019)^37,38^. The recorded IBIs (InterBeat Intervals; time in ms between individual heartbeats) were corrected for artifacts using our custom code (see https://osf.io/78g9s/). We assessed the data quality, resulting in the use of 73 out of 77 participants’ cardiac responses. Twelve IBIs were selected around the feedback: the IBI/heartbeat closest to feedback onset (from now on called *IBI*_*0*_), three IBIs preceding the feedback (*IBI*_*-3*_*, IBI*_*-2*_*, IBI*_*-1*_), and eight IBIs during the feedback exposure (*IBI*_*1*_ to *IBI*_*8*_). By the 8th IBI collected after feedback onset, 75% of the trials had passed the 6 s of feedback exposure (See Supplemental Materials). In accordance with the literature^37,39^, we referenced IBI difference scores to the second IBI preceding the feedback onset (IBI_-2_) for each trial. These referenced IBI difference scores are referred to as delta IBIs throughout the manuscript.

To control for the potential effect of sex on the different speech features, sex was considered as a fixed effect for each individual model prior to statistical inference. However, to make sure our models were parsimonious, we bottom-up tested whether adding *sex* as an independent variable to the model improved each model’s fit. For each dependent variable, we compared models that included and excluded *sex*, and it was only included in the model if it showed to be a significant contributor after comparing models with reducing complexity using *χ*^2^ goodness-of-fit tests within the ‘anova()’ function. The statistical significance level was set to p < 0.05 and in the results section, we describe for each individual model whether *sex* was a significant contributor and thus included.

For the manipulation checks (i.e., IBIs and self-reports) and speech features (i.e., F0, jitter, shimmer, harmonics-to-noise ratio, and speech rate), we used the ‘lme4’^40^ and ‘car’^40,41^ R packages to fit generalized linear mixed models (GLMMs). The IBI model featured delta IBI (referenced to IBI_-2_; relative change of IBI as compared to IBI_-2_ indicating acceleration (i.e., negative delta IBI) and deceleration (i.e., positive delta IBI) of the heart) as a dependent variable with 12 levels (IBI_-3_ to IBI_8_), feedback condition (2 levels; control vs negative feedback condition) as a fixed effect, and the subject as a random intercept. The ANOVA comparison for the model including vs excluding *sex* as a fixed effect showed no significant improvement and *sex* was thus excluded from the model. The valence and arousal models followed a similar structure. Either valence or arousal as the dependent variable on a 7-point Likert scale, having 2 levels of feedback condition (control vs negative feedback) as a fixed effect, and subject as a random intercept. Again, the ANOVA analysis showed no significant contribution of *sex* to these models, and *sex* was thus also excluded from these models as a fixed effect. The models for the speech features were identical to the valence/arousal models, with feedback condition (2 levels; control vs negative feedback, each containing 3 data points per participant) as a fixed effect, and subject as random intercept whilst controlling for sex by including it as a fixed effect if aforementioned method showed it to have a significant contribution to the model, resulting in the (G)LMM formulas of the following structure (in R notation for *‘lme4’*); *DependentVariable* ~ *Condition* + *Sex* + *(1|ID)* or *DependentVariable* ~ *Condition* + *(1|ID)*. Each dependent variable’s specific model is also reported in the results section.

The sum of squares was estimated using the type III approach, and the statistical significance level was set to *p* < 0.05. Follow-up tests with pairwise comparisons of the EMMs (estimated marginal means) were performed with the ‘emmeans’ package^42^, using false discovery rate (FDR) to correct for multiple testing^23^.

## Supplementary Information

Supplementary Information.

## Data Availability

All data and corresponding code are openly available at https://osf.io/78g9s/.
