# Supplementary material for: Acoustic speech features in social comparison: how stress impacts the way you sound
Source: Sci Rep. 2022 Dec 20;12:22022. doi: 10.1038/s41598-022-26375-9 (PMC9767914; doi:10.1038/s41598-022-26375-9)
Supplement: Supplementary file 1 — Supplementary Information. [file 41598_2022_26375_MOESM1_ESM.pdf]

Supplemental Materials

All data and corresponding code are openly available through https://osf.io/78g9s/. Code works out-of-the-box with instructions found in corresponding README.md in osf directory.

Exclusion criteria:

- Other than native Dutch speakers
- Left-handed
- Born before 1970
- Psychology student
- Personal or family history of epilepsy
- Recent neurosurgical procedures
- Pacemaker or other electronic implants
- Inner ear prosthesis
- Metal objects or magnetic objects in the brain or around the head (only removable earrings & piercings are allowed)
- Pregnancy
- Unstable medical condition
- A current depressive episode
- Other psychiatric disorders
- Skin disorder at the level of the head
- Current addiction
- Current substance abuse
- Current use of psychotropic medication
- Eye disease(s)
- Heart, respiratory, or neurological problems
- Did not drink coffee or smoke 2 hours before the start of the experiment
- Dreadlocks

Response Block:

First: Read out loud text “Marloes”:

“Papa en Marloes staan op het station. Ze wachten op de trein. Eerst hebben ze een kaartje gekocht. Er stond een hele lange rij, dus dat duurde wel even. Nu wachten ze tot de trein eraan komt. Het is al vijf over drie, dus het duurt nog vier minuten. Er staan nog veel meer mensen te wachten. Marloes kijkt naar links, in de verte ziet ze de trein al aankomen.”

From: van de Weijer and Slis (1991)

Second: Answer Self-Assessment Manikins (SAMs)

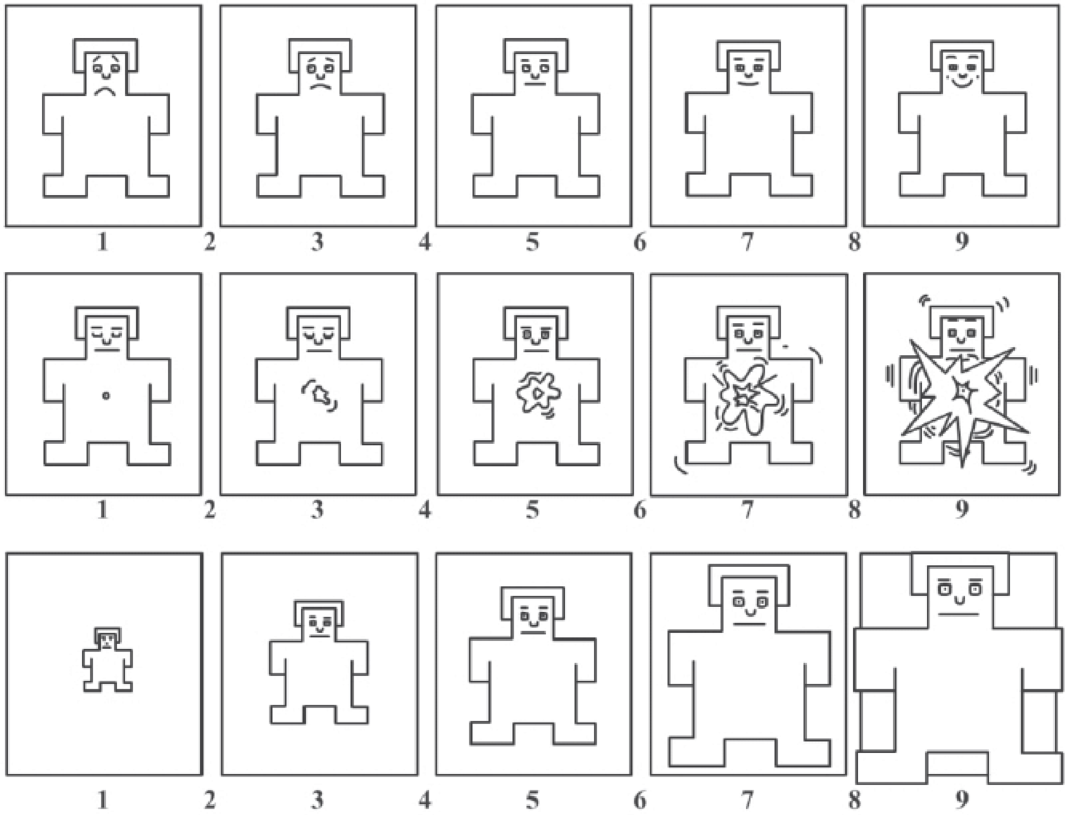

Self-Assessment Manikin (SAM) Scales (Bradley & Lang, 1994)

Third: Read out loud and answer Brief State Rumination Index (BSRI; Marchetti, Mor, Chiorri & Koster, 2018)

Supplemental Figure 1.

Boxplots indicating time passed since feedback onset for each relative delta IBI. Horizontal bars indicate the lower and upper limit, box borders indicate the 25th and 75th percentile, and lines in the boxplot indicate the median. A horizontal dotted line indicates the 6 seconds mark: total feedback exposure time. This shows that 75% of the trials had passed the 6 seconds of feedback exposure at IBI8.

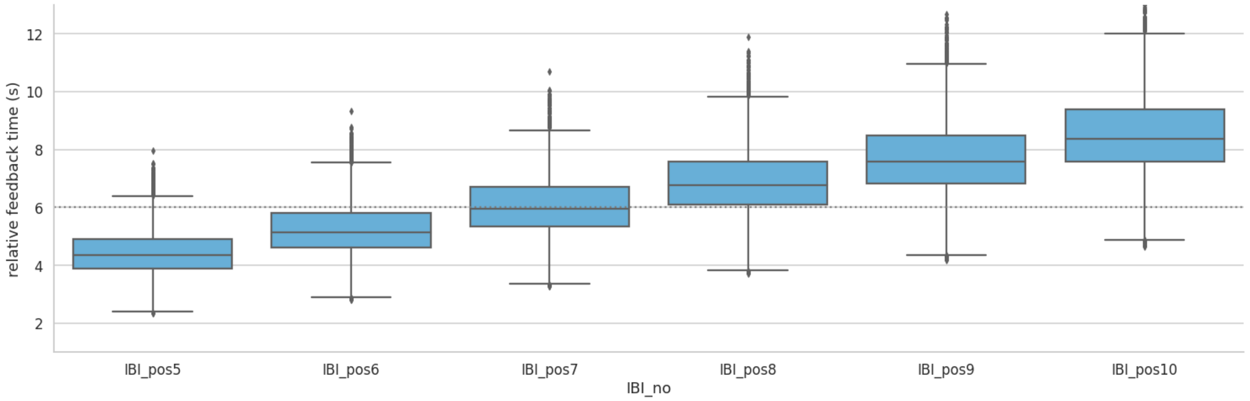

Supplemental Figure 2.

Performance feedback as lines in the colored feedback bar. Blue dots stand for 'group performance', and orange dots stand for 'your performance'. Y-axis shows time over the condition (left two plots are control condition; right two plots are negative condition). Vertical dashed lines show separations for the subblocks (where a response block was executed).

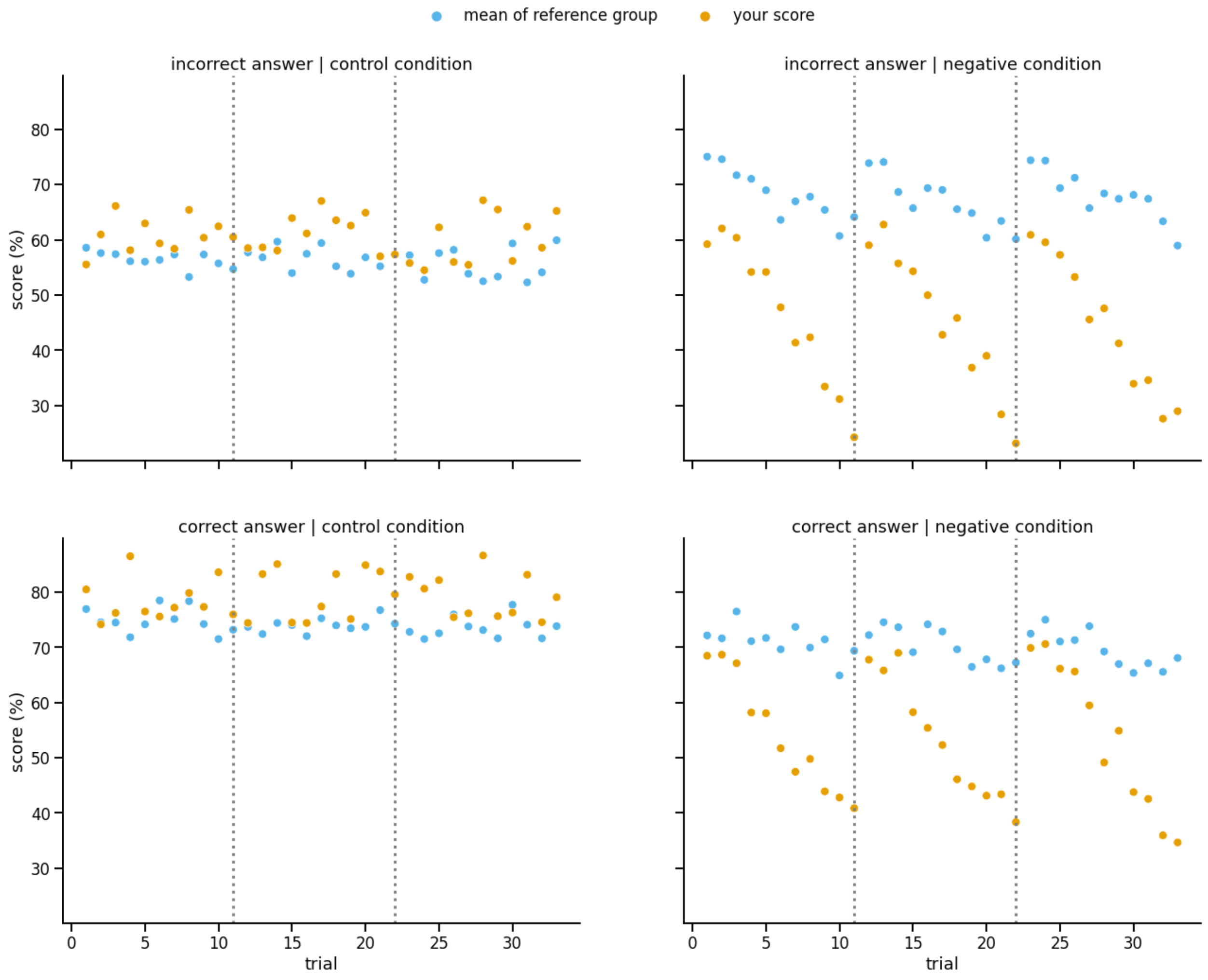

R Packages and session info:

```
> sessionInfo()
R version 4.1.1 (2021-08-10)
Platform: x86_64-w64-mingw32/x64 (64-bit)
Running under: Windows 10 x64 (build 19043)

Matrix products: default

locale:
[1] LC_COLLATE=English_Belgium.1252  LC_CTYPE=English_Belgium.1252    LC_MONETARY=English_Belgium.1252 LC_NUMERIC
[5] LC_TIME=English_Belgium.1252

attached base packages:
[1] stats     graphics  grDevices utils     datasets  methods   base

other attached packages:
 [1] dplyr_1.0.7          tibble_3.1.4          arrow_5.0.0.2         car_3.0-11            ggpubr_0.4.0
 [6] ggplot2_3.3.5        effectsize_0.4.5      effects_4.2-0         carData_3.0-4         lmerTest_3.1-3
[11] reshape_0.8.8        pander_0.6.4          emmeans_1.6.3         lme4_1.1-27.1         yarrr_0.1.5
[16] circlize_0.4.13      BayesFactor_0.9.12-4.2 Matrix_1.3-4         coda_0.19-4           jpeg_0.1-9

loaded via a namespace (and not attached):
 [1] nlme_3.1-152        bit64_4.0.5         insight_0.14.4      numDeriv_2016.8-1.1 tools_4.1.1         backport
 [7] utf8_1.2.2          R6_2.5.1            DBI_1.1.1           colorspace_2.0-2    nnet_7.3-16         withr_2.
[13] tidyselect_1.1.1    bit_4.0.4           curl_4.3.2          compiler_4.1.1      sandwich_3.0-1      bayestes
[19] scales_1.1.1        mvtnorm_1.1-2       pbapply_1.4-3       stringr_1.4.0       digest_0.6.27       foreign_
[25] minqa_1.2.4         rio_0.5.27          pkgconfig_2.0.3     rlang_0.4.11        GlobalOptions_0.1.2 readxl_1
[31] rstudioapi_0.13     shape_1.4.6         generics_0.1.0      zoo_1.8-9           gtools_3.9.2        zip_2.2.
[37] magrittr_2.0.1      parameters_0.14.0   Rcpp_1.0.7          munsell_0.5.0       fansi_0.5.0         abind_1.
[43] lifecycle_1.0.0     stringi_1.7.4       multcomp_1.4-18     MASS_7.3-54         plyr_1.8.6          grid_4.1
[49] parallel_4.1.1      forcats_0.5.1       crayon_1.4.1        lattice_0.20-44     haven_2.4.3         splines_
[55] hms_1.1.0           pillar_1.6.2        boot_1.3-28         estimability_1.3    ggsignif_0.6.3      codetool
[61] glue_1.4.2          mitools_2.4         data.table_1.14.0   vctrs_0.3.8         nloptr_1.2.2.2      cellrang
[67] MatrixModels_0.5-0  gtable_0.3.0        purrr_0.3.4         tidyr_1.1.3         assertthat_0.2.1    datawiza
[73] openxlsx_4.2.4      xtable_1.8-4        broom_0.7.9         survey_4.1-1        rstatix_0.7.0       survival
[79] TH.data_1.1-0       ellipsis_0.3.2
```

Corresponding feature names as described in GeMAPS (Eyben et al., 2016)

Jitter = 'jitterLocal_sma3nz_amean',
Shimmer = 'shimmerLocaldB_sma3nz_amean',
F0 = 'F0semitoneFrom27.5Hz_sma3nz_amean',
HNR = 'HNRdBACF_sma3nz_amean',
Voiced Segments per Second = 'VoicedSegmentsPerSec',
Voiced Segment Length = 'MeanVoicedSegmentLengthSec'
